# Supplementary material for: Single-cell transcriptome profiling of the stepwise progression of head and neck cancer
Source: Nat Commun. 2023 Feb 24;14:1055. doi: 10.1038/s41467-023-36691-x (PMC9958029; doi:10.1038/s41467-023-36691-x)
Supplement: Supplementary file 1 — Supplementary Information [file 41467_2023_36691_MOESM1_ESM.pdf]

SUPPLEMENTARY INFORMATION

Supplementary Methods

Processing and clustering of scRNA-seq data

The raw data of the gene expression matrix was filtered using the "Seurat" R package with the following criteria of the cells with > 200 genes and < 8000 genes, < 10% of mitochondrial gene expression in UMI counts, and the genes expressed in > 0.1% cells. The batch effects of the samples were corrected using 'RunRunHarmony' in the "Harmony" R package, dimension reduction was performed using UMAP with the 'RunUMAP' function (dims = 1:50), and the clustering was performed using a shared-nearest neighbor graph algorithm 'FindClusters' function (resolution = 0.2). Subtyping of the malignant cells, cancer-associated fibroblasts (CAFs), and T cells were performed using the following parameters. For malignant cells, dims = 1:20, lambda = 1, theta = 0, and resolution = 0.05; for CAFs, dims = 1:10, lambda = 1, theta = 0, and resolution = 0.2; for T cells, dim = 1:50, lambda = 1, theta = 2, and resolution = 0.2.

Cell typing, deconvolution, and gene set analyses

Cell typing was performed by manually examining the expression levels of the canonical marker genes for malignant cells (*KRT14*, *KRT17*, *KRT6A*, *KRT5*, *KRT19*, *KRT8*, *KRT16*, *KRT18*, *KRT6B*, *KRT15*, *KRT6C*, *KRTCAP3*, *EPCAM*, *SFN*), fibroblasts (*FAP*, *PDPN*, *COL1A2*, *DCN*, *COL3A1*, *COL6A1*), myocytes (*ACTA1*, *ACTN2*, *MYL2*, *MYH2*), T cells (*CD2*, *CD3D*, *CD3E*, *CD3G*), B/Plasma cells (*SLAMF7*, *CD79A*, *BLNK*, *FCRL5*), macrophages (*CD14*, *CD163*, *CD68*, *FCGR2A*, *CSF1R*), dendritic cells (*CD40*, *CD80*, *CD83*, *CCR7*), mast cells (*CMA1*, *MS4A2*, *TPSAB1*, *TPSB2*) and endothelial cells (*PECAM1*, *VWF*, *ENG*). Deconvolution of the bulk RNA-Seq data s was performed using “MuSiC” R package [1].

The proliferation index score was calculated as the sum of the S scores and G2M scores using the 'CellCycleScoring' function in the Seurat R package. The scores for 'tumor-infiltrating lymphocyte (TIL)' and 'dysfunctional T cell' were calculated as the average expression values of the marker genes, respectively [2, 3].

Identification of recurrent CNAs

We developed a CNA calling method to detect the recurrent CNAs, considering the different cell proportions in each sample. We determined the tissue type-specific CNAs and the sample-specific CNAs, respectively. Tissue-specific CNAs were determined by comparing the frequencies of the cells with CNAs in each tissue (*i.e.*, LP vs. NL and CA/LN vs. LP) with a cutoff of the fold difference of the cell proportion with CNAs greater than 10 % and Fisher's exact test (P < 0.001) between the tissues. In

addition, sample-specific CNAs of the epithelial cells were determined with a cutoff of a cell proportion with CNA greater than 5 %. Then, we defined the recurrent CNAs as the genes observed in at least two samples with overlap between the tissue-specific and the sample-specific CNAs.

Interdependent ligand-receptor interaction analysis

Data for putative ligand-receptor interactions were obtained from a previous study [4]. First, the average expression levels for each ligand-receptor pair were estimated in the malignant cells and the fibroblasts across the tissue types of NL, LP, and CA/LN, respectively. Then, to determine the interdependent ligand-receptor pairs between CAFs and malignant cells, the ligand-receptor pairs were further filtered by the following criteria. (1) The ligands were expressed, but their receptors were not expressed in CAF (ligand *vs.* receptor, fold difference of average expression > 0.5). (2) The receptors were expressed, but their ligands were not expressed in the malignant cells (receptor *vs.* ligand, fold difference of average expression > 0.5). (3) The ligands were expressed in LP than NL (LP vs. NL, fold difference of average expression > 0.1). Because the basal expression levels of the ligands and the receptors in tumor tissues (CA and LN) were substantially higher than those in non-tumor tissues (NL and LP), we applied different cutoffs of the fold difference for each cell type.

Pseudo-time trajectory analysis

Pseudo-time trajectory analyses were performed using Monocle v2 in the R package [5]. UMI count matrices were used to create a CellDataSet object with default parameters. The T cell clusters were identified using the 'FindVariableFeatures' function of the Seurat R package. Gene expression changes according to the pseudo-times were evaluated using the differentialGeneTest function. To construct UMAP-based single-cell state transitions, we applied single-cell trajectory analysis for fibroblasts using the Monocle v3 [6] R package. Dimension reduction and cell clustering were performed using the 'reduce_dimension,' and 'cluster_cell' (k = 50) functions.

Public data analysis

Public data sets for scRNA-Seq of HNSCC patients (GSE103322 and GSE164690) were obtained from Gene Expression Omnibus. After data scaling for malignant cells, the malignant clusters, CC0-CC5, were predicted using the classifiers by applying the nearest template prediction (NTP) algorithm.

Cell lines

FaDu cells were obtained from American Type Culture Collection (Manassas, VA, USA) and cultured in Minimum Essential Medium (MEM; Welgene, Gyeongsangbuk-do, South Korea). MSKQLL1, SCCQLL1, SCC15, and SCC25 were kindly provided by Prof. Se-Heon Kim (Yonsei University, Korea). MSKQLL1, SCC15, and SCC25 cells were maintained in Dulbecco's modified Eagle's Medium/Nutrient

Mixture F-12 (DMEM/F12; Welgene). SCCQLL1 were cultured in Minimum Essential Medium (MEM; Welgene). All growth media were supplemented with 10 % fetal bovine serum, and cells were cultured at 37 °C in a humidified atmosphere containing 5 % CO_2.

Immunohistochemistry

Immunohistochemistry was performed to analyze the expression of galectin-7 (Abcam, ab206435, 1:100). Paraffin tissue sections were briefly deparaffinized with xylene and rehydrated through alcohol and washed in distilled water. After H_2O_2-induced inactivation for endogenous peroxidase activity and antigen retrieval in pepsin (Dako, Carpinteria, CA) at 37 °C for 30 min, the tissue sections were incubated with the primary antibody for LGALS7B overnight at 4 °C. After washing, signals were detected with 3,3-diaminobenzidine tetrahydrochloride, and the sections were counterstained with hematoxylin.

For immunofluorescence staining, paraffin sections were blocked with 5% normal goat serum solution before being incubated overnight at 4 °C with the following primary antibodies: anti-COL1A1 (Santa Cruz Biotechnology, sc-293182, 1:40); anti-CD44 (Abcam, ab189524, 1:1,000); anti-TP63 (Abcam, ab97865, 1:200). After several washes in PBS-T, tissue sections were incubated for 2 hours at room temperature with the following secondary antibodies; Fluorescein (FITC) conjugated anti-rabbit IgG antibody (Jackson Immunoresearch); Cy3 conjugated anti-mouse IgG antibody (Jackson Immunoresearch). Nuclei were stained with 4',6-diamidino-2-phenylindole (DAPI, Sigma-Aldrich). Immunofluorescent images were taken from each slide using LSM 710 confocal microscope (Carl Zeiss).

siRNA-mediated knockdown

The siRNAs targeting *COL1A1* and *CD44* were purchased from Genolution Pharmaceuticals (Seoul, South Korea), and the siRNAs targeting *TP63, ATP1B3,* and negative control were purchased from Dharmacon (SMARTpool: ON-TARGETplus siRNAs). Cells were transfected with siRNA (50 nM) using RNAiMAX (Invitrogen, CA, USA).

Cell viability, migration, and invasion assays

Cells were seeded at a density of 3×10^3 cells per well in 96-well plates. At 24 h post-seeding, cells were transfected with siRNA at various times. Cell viability was measured by using the Cell Counting Kit (CCK-8; Dojindo, Tokyo, Japan) according to the manufacturer's instructions. Each experiment was repeated in triplicate.

Cell migration and invasion assays were performed in a 6.5 mm insert with 8.0 µm polyethylene terephthalate membranes in 24-well plates (SPLInsert™ Hanging, SPL Life Sciences). For the migration assay, cells were transfected with siRNAs targeting *TP63*, *ATP1B3*, or negative control. After 24 h, the

transfected cells (7×10^4) were seeded in a top chamber of 150 µl of serum-free medium. The culture medium with 10% FBS (800 µl) was added to the bottom chamber, and the cells were incubated for 18 h. For cell invasion assay, the upper surface of the membrane was pre-coated with 10 µg/ml fibronectin (Sigma Aldrich, F1141) in PBS overnight at 4°C. 1×10^5 cells/well were seeded on top of the chamber of 150 µl of serum-free medium. The culture medium with 10% FBS (800 µl) was added to the bottom chamber, and the cells were incubated for 24 h. Then, the migrated or invaded cells were fixed with 4% paraformaldehyde phosphate buffer solution (Wako Pure Chemical Industries, 163-20145) for 5 min and stained with 0.1% crystal violet (Sigma Aldrich, C0775) in 20 % methanol solution for 15 min. Non-migrated cells were removed with a cotton swab, and then the numbers of migrated or invaded cells were counted in the random fields under EVOS M7000 Cell Imaging Systems (x200 magnification, Thermo Fisher Scientific). The bound crystal violet was eluted by adding 400 µl of 10 % acetic acid solution (Sigma Aldrich, 45754) into each insert and shaken for 10 min. The eluent on inverted transwells was transferred to a 96-well microplate, and the absorbance at 590 nm was measured using an Epoch Microplate Spectrophotometer (BioTek Instruments). Both experiments were repeated in triplicate, independently.

Co-culture of MSKQLL1 cells and the patient-derived CAFs were implemented using the indirectly co-culture transwell system [6.5 mm insert with 8.0 µm polyethylene terephthalate membranes in 24-well plates, SPLInsert™ Hanging, SPL Life Sciences]. The cells were transfected with si*CD44* or si*COL1A1*, respectively. 1×10^5 of the malignant cells were seeded in the top chamber with serum-free medium (150 µl), and 1×10^5 of the patient-derived CAFs were seeded in the bottom chamber with culture medium (800 µl, 10% FBS) and incubated for 18 h.

Sphere formation assays

FaDu cells were transfected with 50 nM of the siRNAs targeting *TP63*, *ATP1B3*, or negative control. After 24 h transfection, the single-cell suspension of the transfected cells (3×10^3 cells/35 µl/drop) in a complete culture medium was placed on the inner side of a 96-well hanging drop plate (SPL Life Sciences). After 48 h incubation, the spheroids were harvested from the hanging drop plate by pipetting 100 µl of PBS. The spheroids were then washed with PBS and stained using the LIVE/DEADTM Viability/Cytotoxicity Kit (Thermo Fisher Scientific, L3224) by incubating with calcein AM and Ethidium homodimer-1 (EthD-1) at 37°C for 30 min. A confocal microscope (Nikon A1R, Japan, x20 objective, n = 8) was used for imaging, and z-section imaging was performed.

Western blotting

Cells were lysed with RIPA buffer (25 mM Tris-HCl pH 7.6, 150 mM NaCl, 1 % NP-40, 1 % sodium deoxycholate, 0.1% SDS) (Sigma) containing a protease and phosphatase inhibitor cocktail (Thermo Fisher Scientific, 78440) on ice for 30 min. Protein samples were separated by SDS-PAGE and transferred to a polyvinylidene difluoride (PVDF) membrane. Each membrane was blocked with 5% skim milk for 1 h at room temperature and incubated overnight with primary antibody (1:1,000) at 4°C. After washing with 0.1% Tween-20 (Sigma-Aldrich, P1379) in Tris-buffered saline (Sigma-Aldrich, T8912), the membranes were incubated with an HRP-conjugated secondary antibody (1:5,000). Proteins were visualized using ECL reagents (GE Healthcare Life Sciences, RPN2235) and detected with ImageQuant™ LAS 4000 (FujiFilm, Tokyo, Japan). Antibodies were obtained from several sources, including anti-CD44 (Cell Signaling Technology, 3570), anti-COL1A1 (Cell Signaling Technology, 72026), anti-GAPDH (Cell Signaling Technology, 5174), anti-TP63 (Abcam, ab97865), anti-ATP1B3 (Santa Cruz Biotechnology, sc-135998), anti-alpha Tubulin (Merck Millipore, CP06).

RT-PCR

Total RNA was extracted using TRIzol reagent (Thermo Fisher Scientific, 15596026). cDNAs were synthesized with 1 µg of total RNAs and ReverTraAce qPCR RT Master Mix (TOYOBO, Osaka, Japan, FSQ-201) according to the manufacturer's instructions. The transcripts were qualitatively measured using a SYBR Green Realtime PCR Master Mix (TOYOBO, QPK-201) according to the manufacturer's directions and run on a StepOnePlus Real-Time PCR system (Applied Biosystems). Relative expression levels (Rq**)** of the indicated genes were compared with the GAPDH expression levels of their corresponding samples using the ΔΔCt method (StepOne Plus Software v2.3; Applied Biosystems). Primers were obtained from several sources, including *GAPDH* (Qiagen, Hs_QT00079247), *CXCL8* (Origene, HP200551), *LSGALS7* (Sino Biological, HP101538), *COL1A1* (Qiagen, Hs_QT00037793), *Foxp3* (Qiagen, Mm_QT00138369), and *Gapdh* (Qiagen, Mm_QT01658692).

References for Supplementary Methods

1. Wang X, Park J, Susztak K, Zhang NR, Li M. Bulk tissue cell type deconvolution with multi-subject single-cell expression reference. *Nature Communications* **10**, 380 (2019).

2. Danaher P*, et al.* Gene expression markers of Tumor Infiltrating Leukocytes. *J Immunother Cancer* **5**, 18 (2017).

3. Li H*, et al.* Dysfunctional CD8 T Cells Form a Proliferative, Dynamically Regulated Compartment within Human Melanoma. *Cell* **176**, 775-789 e718 (2019).

4. Ramilowski JA*, et al.* A draft network of ligand-receptor-mediated multicellular signalling in human. *Nat Commun* **6**, 7866 (2015).

5. Qiu X*, et al.* Reversed graph embedding resolves complex single-cell trajectories. *Nat Methods* **14**, 979-982 (2017).

6. Cao J*, et al.* The single-cell transcriptional landscape of mammalian organogenesis. *Nature* **566**, 496-502 (2019).

Supplementary Figures

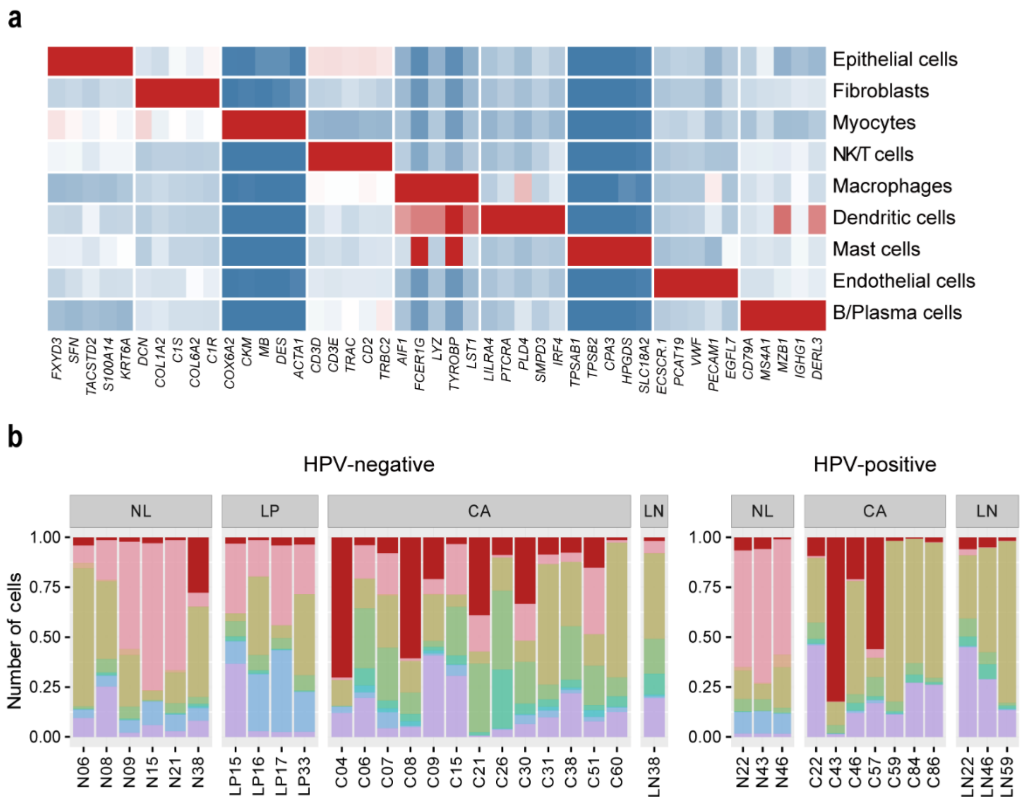

Supplementary Fig. 1. Expression of cell type cluster markers and distribution of cell types.

a. A heatmap shows the expression levels of the five top-ranked marker genes for each cell type. **b**. Distributions for the proportion of cell types in individual samples are shown.

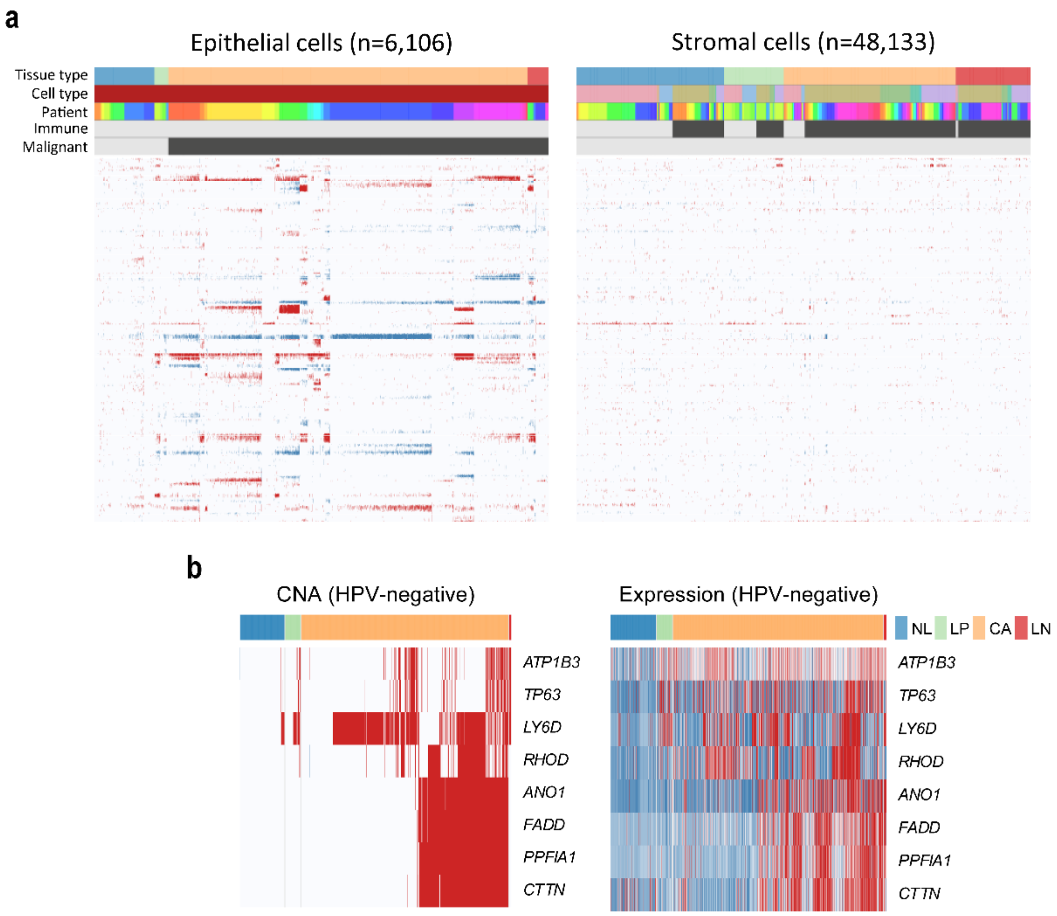

Supplementary Fig. 2. DNA copy number aberrations during HNSCC progression.

a. Heatmap showing the copy number aberrations (CNAs) inferred from the single-cell RNA-seq data.

b. Heatmaps show the CNAs (*left*) and expression levels (*right*) of genes with CNA-dependent transcriptional deregulation in LP or CA (LP; *ATP1B3*, *TP63*, and *LY6D*, CA; *RHOD*, *ANO1*, *FADD*, *PPFIA1*, and *CTTN*) during HNSCC progression.

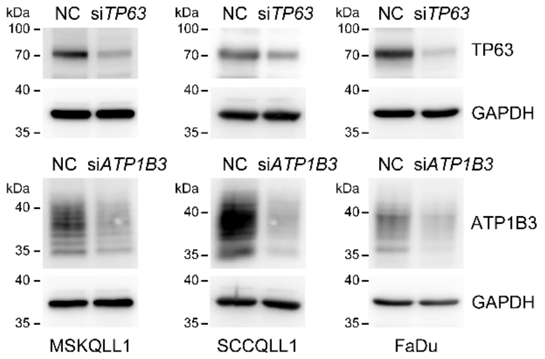

Supplementary Fig. 3. Effect of *TP63* or *ATP1B3* knockdown in HNSCC cells.

The MSKQLL1, SCCQLL1, and FaDu cells were treated with siRNAs targeting *TP63*, *ATP1B3,* or non-target control (NC). Cells were harvested with RIPA buffer and subjected to Western blot to detect TP63 or ATP1B3 and GAPDH. Source data are provided as a Source Data file.

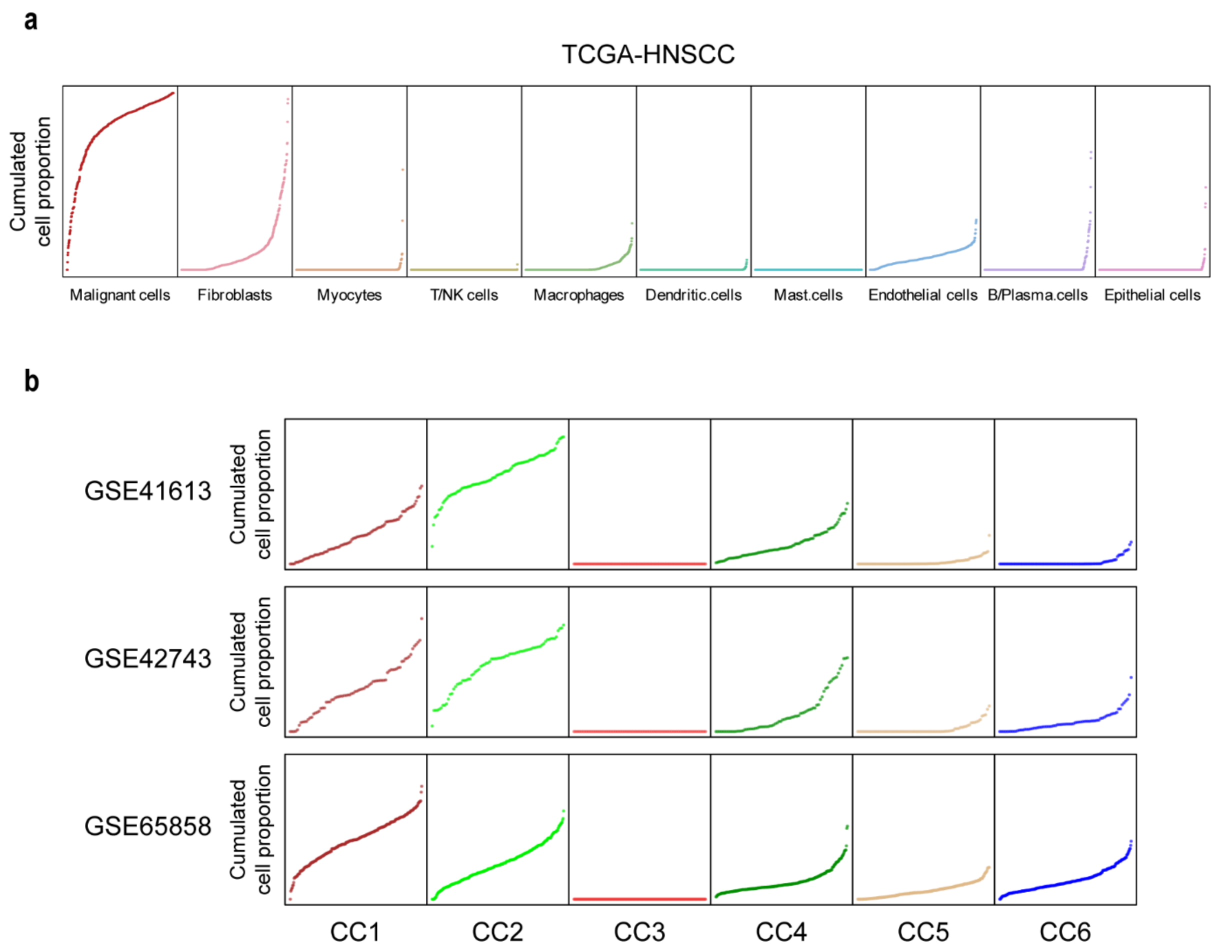

Supplementary Fig. 4. Deconvolution analysis for cell types and malignant cell clusters.

a. Cumulative proportions of each cell type as measured by deconvolution analysis in the TCGA-HNSCC data set are shown. **b.** Cumulative proportions of each malignant cluster as measured by deconvolution analysis in GSE41613, GSE42743, and GSE65858 are shown. Samples are ordered according to the proportion of each cluster.

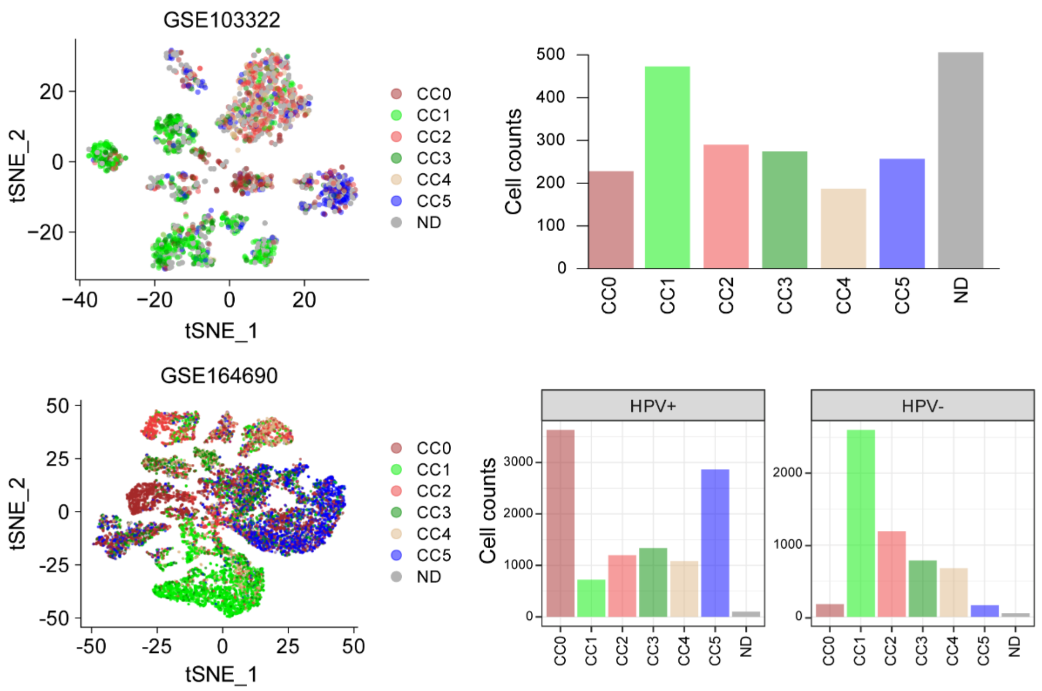

Supplementary Fig. 5. Nearest template prediction analysis to classify malignant cell clusters. The malignant cell clusters (CC0-CC5) are indicated in t-SNE plots (*left*) and cell counts are shown across the malignant cell clusters (*right*) in GSE103322 (*top*) and GSE164690 (*bottom*) data sets. Each malignant cell was classified into malignant cell clusters using NTP algorithm.

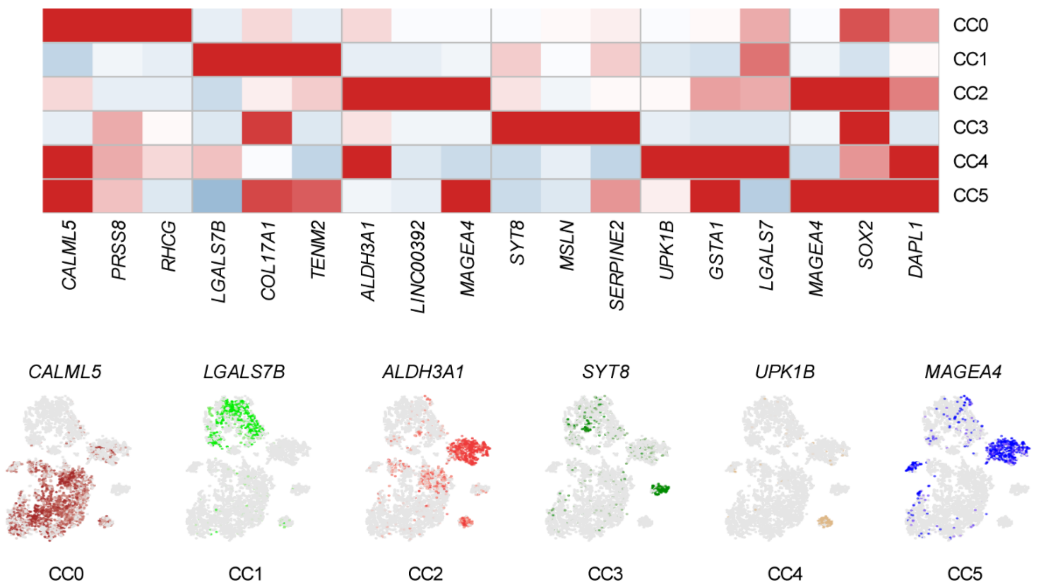

Supplementary Fig. 6. Malignant cell clusters of HNSCC.

Expression levels of top-ranked marker genes for each cell type are shown in a heatmap (*top*) and t-SNE plots (*bottom*).

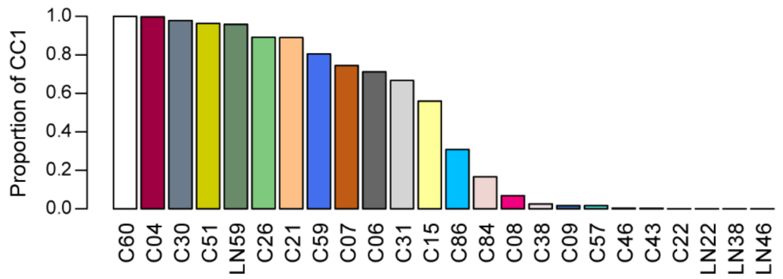

Supplementary Fig. 7. Distribution of CC1 malignant cluster in HNSCC patients.

A bar plot showing the proportion of CC1 clusters in each of the HNSCC samples.

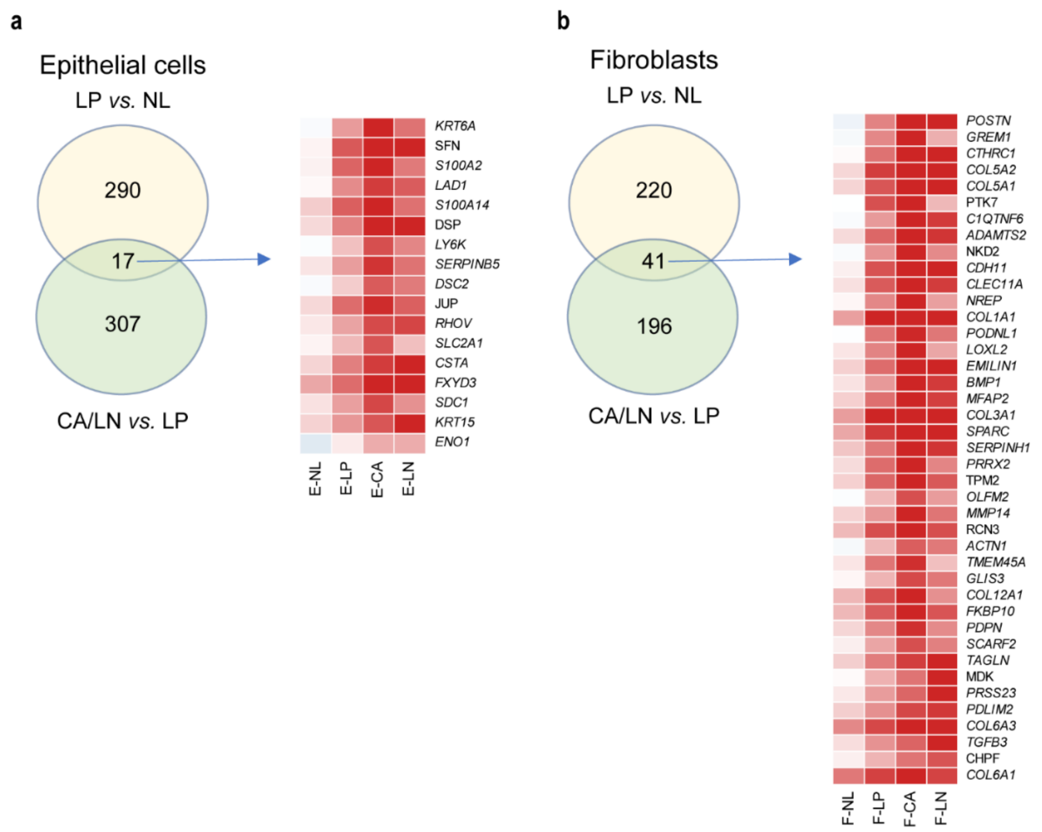

Supplementary Fig. 8. Identification of the genes with stepwise expression in epithelial cells or fibroblasts.

The stepwisely expressed genes during HNSCC progression (LP *vs.* NL, CA/LN *vs.* LP; Permuted two-sided Student's t-test P < 0.001, fold difference > 0.5) in epithelial cells **(a)** and fibroblasts **(b)** are shown.

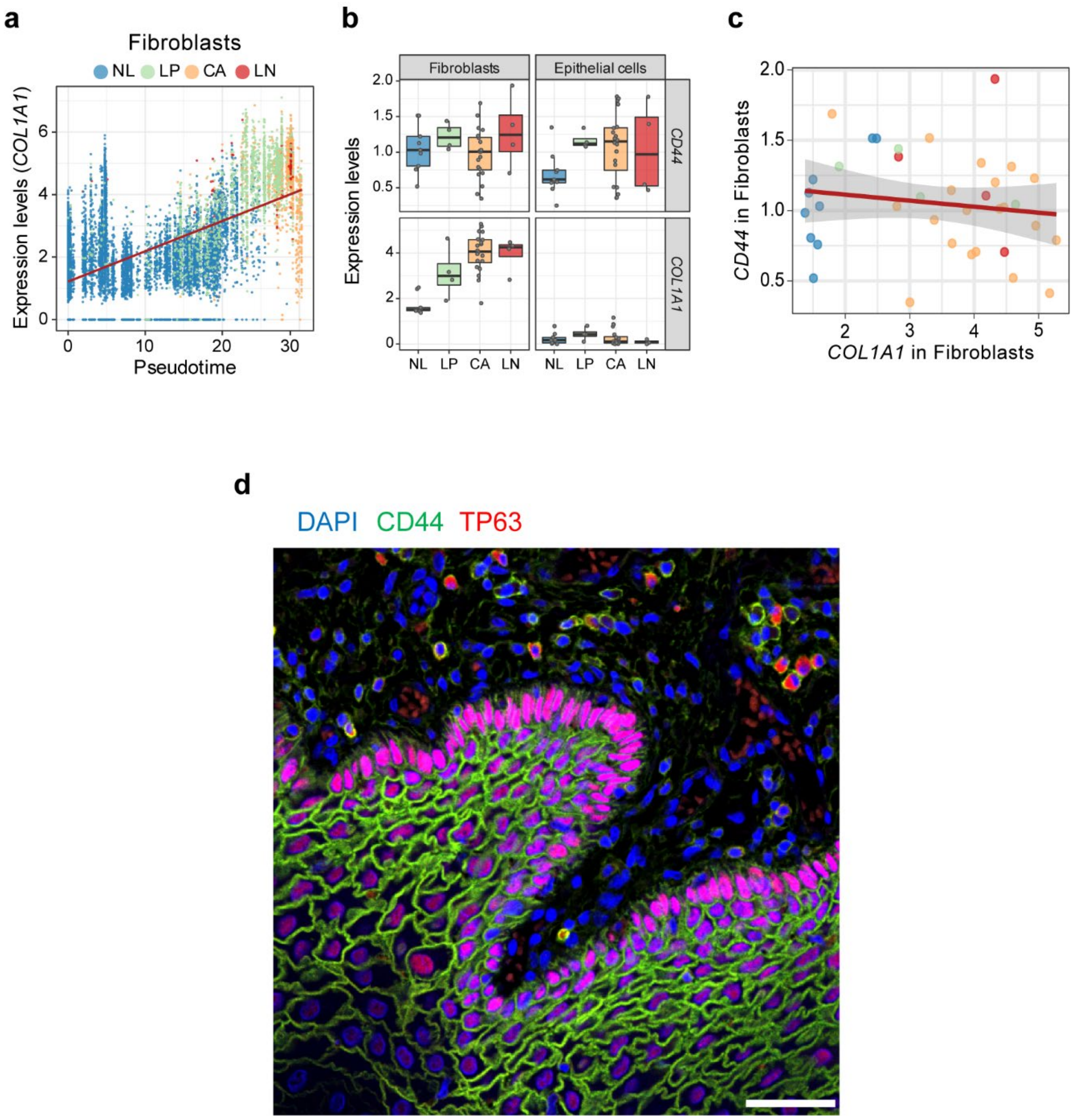

Supplementary Fig. 9. Fibroblast-derived *COL1A1* expression interacts with *CD44* in malignant cells.

a. Correlations between pseudo-times and *COL1A1* expression levels in fibroblasts are shown. **b.** The expression levels of *CD44* and *COL1A1* in fibroblasts (n=12,336) and epithelial cells (n=6,106) according to the tissue types are shown. Box plots show median (center line), the upper and lower quantiles (box) and the range of the data (whiskers). **c.** Correlations between expression levels of *CD44* and *COL1A1* in fibroblasts are shown. The gray shading represents 95% confidence interval (CI). **d.** An immunohistochemical image shows the expression of CD44 (*green*) and TP63 (*red*) in malignant cells. *n* = 3, Scale bar, 50µm.

a

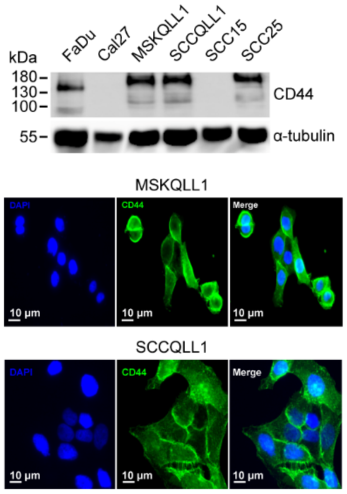

b

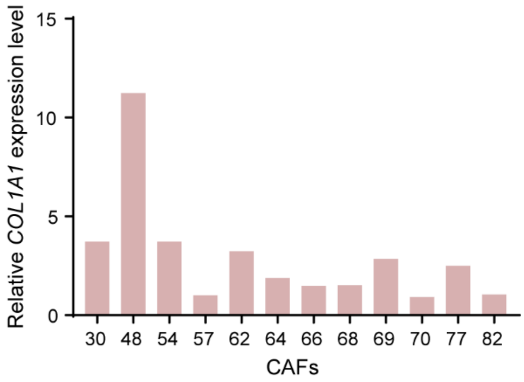

Supplementary Fig. 10. Validation of CD44 expression in malignant cells and COL1A1 in CAFs.

a. Western blots show the expression levels of CD44 in different HNSCC cell lines (*top*). Immunostaining shows the expression of CD44 in MSKQLL1 and SCCQLL1 cell lines, respectively (*bottom*). **b.** Relative COL1A1 expression in the different CAF cells is measured by real-time RT-PCR. Source data are provided as a Source Data file.

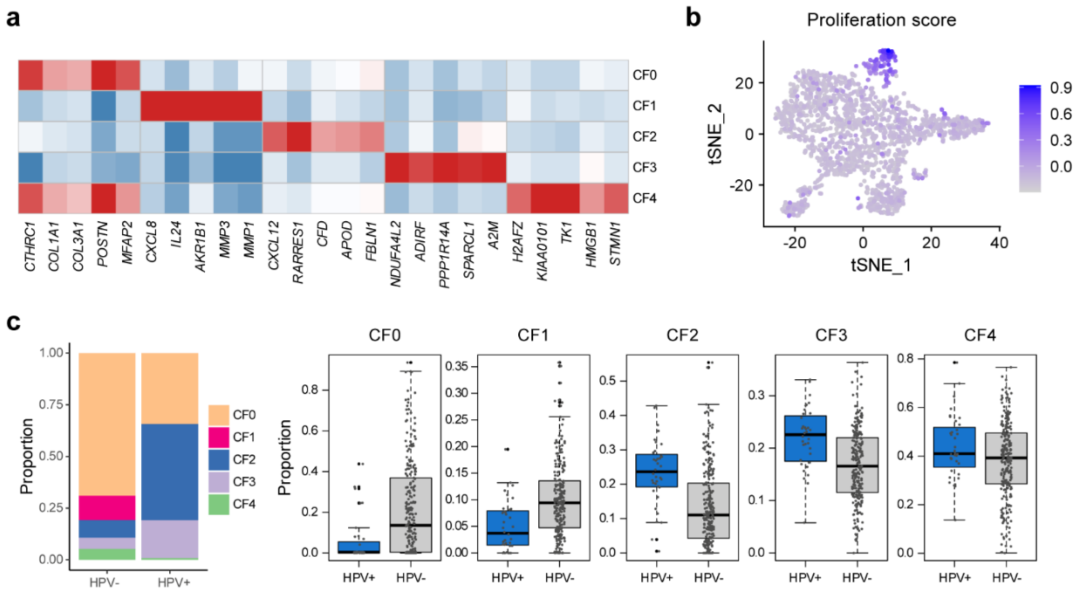

Supplementary Fig. 11. CAF cell clusters of HNSCC.

a. A heatmap shows the expression levels of marker genes for each CAF cell cluster. **b.** Proliferation scores in CAF cells are shown in a t-SNE plot. **c.** A bar plot shows the proportion of CAF cell clusters (CF0-CF4) according to the HPV infection status in scRNA-Seq data (*left*). Deconvoluted proportions according to the HPV infection status for each CAF cluster in TCGA-HNSCC data are shown (*right*). Box plots show median (center line), the upper and lower quantiles (box) and the range of the data (whiskers).

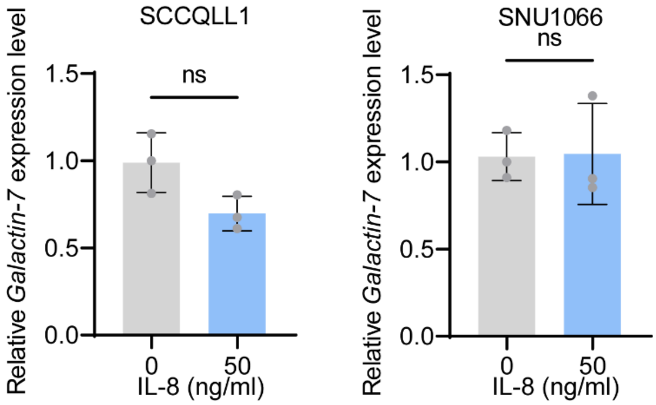

Supplementary Fig. 12. Effect of IL-8 treatment on galectin-7 expression.

Effects of IL-8 (50 ng/mL) treatment in galectin-7 expression are shown in SCCQLL1 and SNU1066 cells. N.S, not significant. n = 3 biological independent experiments. Data are shown as mean ± SD. Source data are provided as a Source Data file.

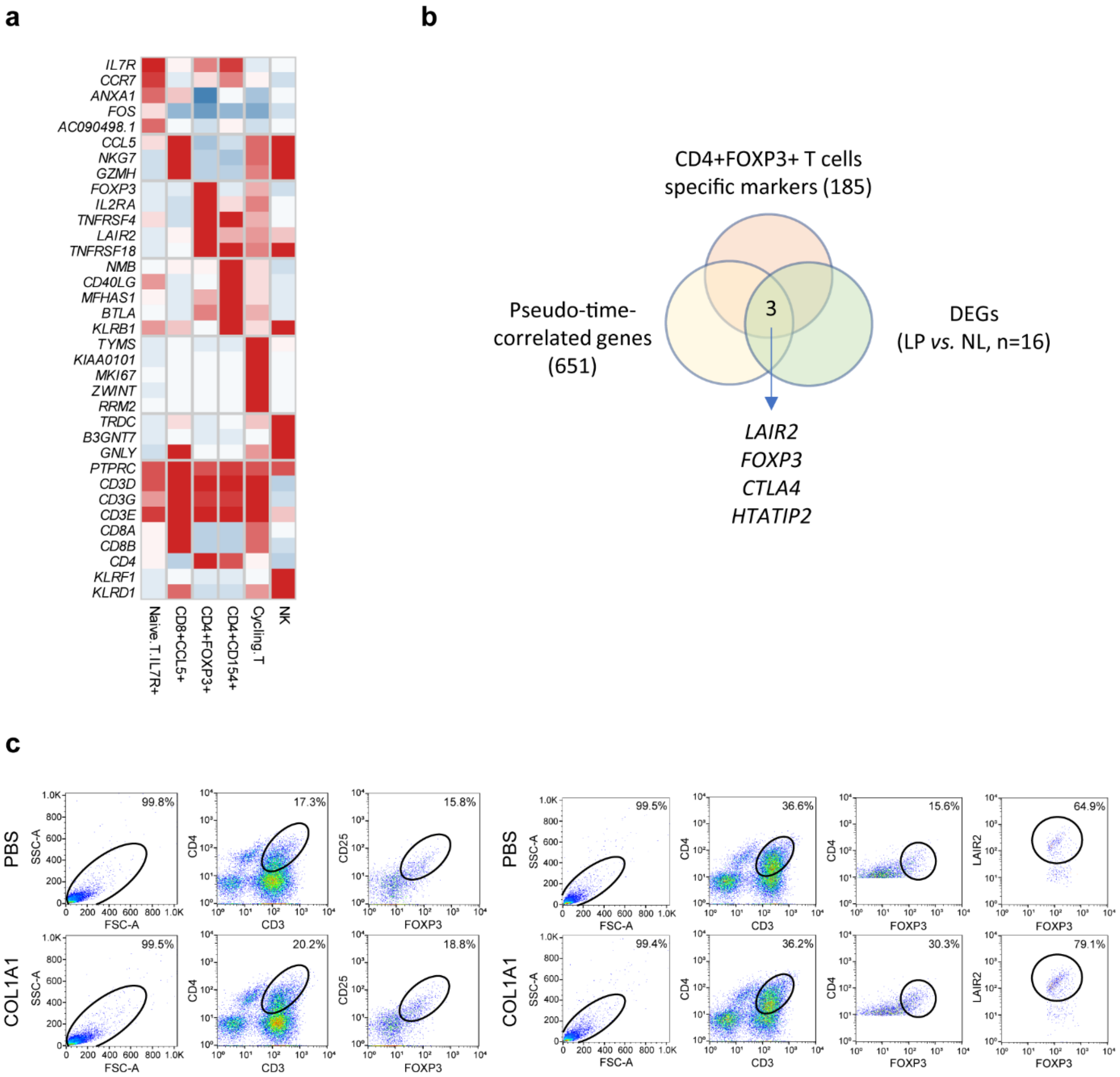

Supplementary Fig. 13. T/NK cell clusters of HNSCC and identification of the potential driver genes for Treg transformation.

a. A heatmap shows the expression levels of marker genes for each T/NK cell cluster and known cell type markers, including *PTPRC* (*CD45*), *CD3D*, *CD3G*, *CD3E*, *CD8A*, *CD8B*, *CD4*, *KLRF1*, and *KLRD1*. **b.** A Venn diagram shows the overlapped genes among the marker genes for Tregs (CD4+FOXP3+ T cells), pseudo-time-correlated genes, and the DEGs for LP *vs.* NL. **c.** Flow cytometry gating strategy used to define LAIR2+FOXP3+ (*left*) and LAIR2+FOXP3+CD25+ (*right*) Treg cell population from patient PBMC is shown. The PMBCs were cultured on Collagen type 1A1 (10 µg/cm^2) coated (*top*) or non-coated plate (*bottom*) for 36 hr.

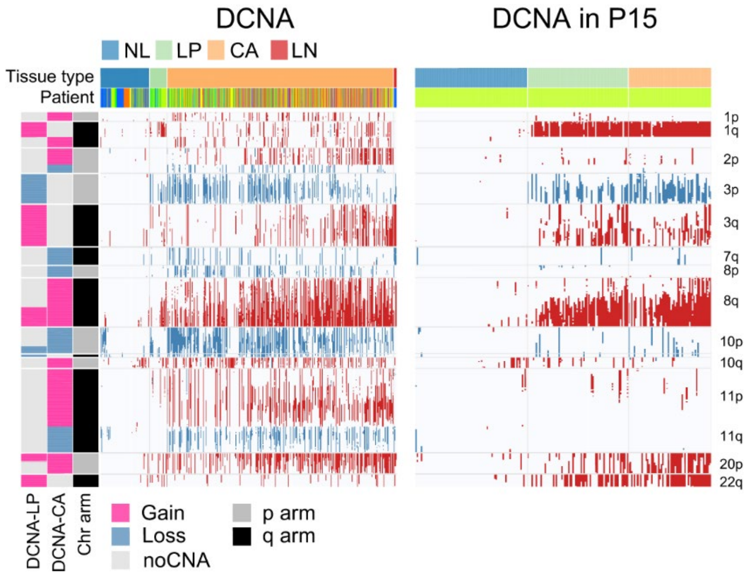

Supplementary Fig. 14. Differential DNA copy number aberrations during HNSCC progression.

Heatmaps show the stepwisely differential CNAs during HNSCC in all epithelial cells (*left*) and cells of patient P15 (*right*). The DCNAs for LP (DCNA-LP) and CA (DCNA-CA) tissue are indicated in the left bar.

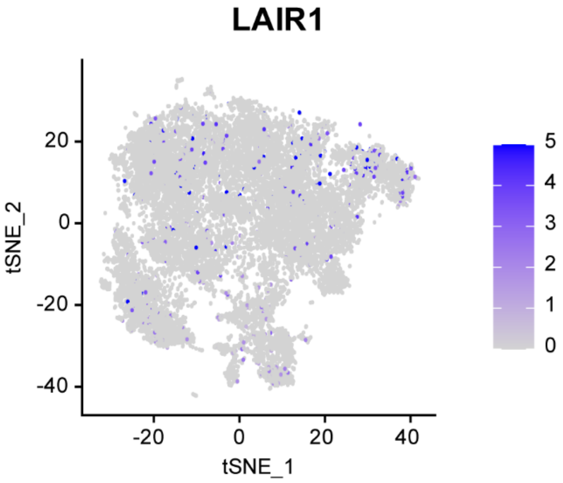

Supplementary Fig. 15. *LAIR1* is less expressed in T/NK cells.

A t-SNE plot shows the expression levels of *LAIR1* in T/NK cells.

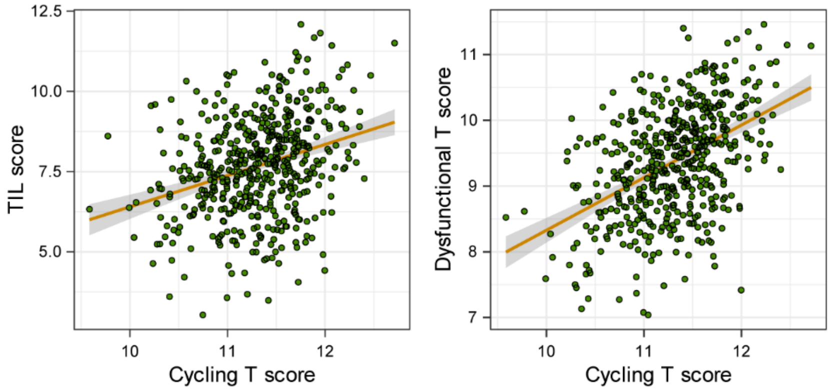

Supplementary Fig. 16. Correlation of Cycling T cells with TIL and dysfunctional T cell signatures in TCGA-HNSCC.

Cycling T score correlated with TIL score (*left*) and dysfunctional T score (*right*) in TCGA-HNSCC data. The gray shading represents 95% confidence interval (CI). Cycling T cell, TIL, and dysfunctional T cell scores are estimated as mean expression values of their marker genes, respectively.

Supplementary Tables

Supplementary Table 1. Patients and samples included in scRNA-Seq data.

Patient ID	Tumor subsite	HPV infection	Tumor stage	Sample ID			
				NL	LP	CA	LN
**P4**	OC	Negative	T2N2a			C04	
**P6**	OC	Negative	T2N1	N06		C06	
**P7**	OC	Negative	T2N0			C07	
**P8**	OP	Negative	T1N0	N08		C08	
**P9**	OP	Negative	T2N0	N09		C09	
**P15**	OC	Negative	T1N0	N15	LP15	C15	
**P16**	OC	Negative			LP16		
**P17**	OC	Negative			LP17		
**P21**	OC	Negative	T4aN1	N21		C21	
**P22**	OP	Positive	T2N2	N22		C22	LN22
**P26**	OC	Negative	T2N0			C26	
**P30**	OC	Negative	T2N0			C30	
**P31**	OC	Negative	T2N2			C31	
**P33**	OC	Negative			LP33		
**P38**	HP	Negative	T4aN3b	N38		C38	LN38
**P43**	OP	Positive	T3N0	N43		C43	
**P46**	OP	Positive	T2N1	N46		C46	LN46
**P51**	OC	Negative	T2N1			C51	
**P57**	OP	Positive	T1N0			C57	
**P59**	OP	Positive	T3N2b			C59	LN59
**P60**	OC	Negative	T4aN0			C60	
**P84**	OP	Positive	T2N1			C84	
**P86**	OP	Positive	T2N0			C86	

OC, oral cavity; OP, oropharynx; HP, hypopharynx; NL, normal; LP, leukoplakia; CA, primary cancer; LN, metastasized lymph nodes.

Supplementary Table 2. Univariate and multivariate analysis for CC1 cluster in HPV-negative HNSCC cohorts.

Variable		Univariate analysis (OS)			Multivariate analysis (OS)		
		HR	95% CI	*P*[a]	HR	95% CI	*P*[a]
**GSE41613 cohort**							
**CC1 %**	Low (≤70th percentile)						
	High (>70th percentile)	2.58	1.47-4.52	**0.001***	2.68	1.50-4.77	**0.001***
**Age**	≤55						
	>55	0.74	0.42-1.29	0.281	0.81	0.46-1.42	0.456
**Sex**	Female						
	Male	1.09	0.60-1.29	0.281	1.31	0.72-2.42	0.377
**GSE42743 cohort**							
**CC1 %**	Low (≤70th percentile)						
	High (>70th percentile)	2.24	1.18-4.25	**0.013***	2.33	1.22-4.44	**0.011***
**Age**	≤55						
	>55	1.29	0.68-2.44	0.431	1.14	0.60-2.18	0.692
**Gender**	Female						
	Male	0.68	0.34-1.36	0.270	0.62	0.31-1.26	0.190

[a]Statistical tests were performed with log-rank test.

Supplementary Table 3. Reagents and resources used in the experiments.

REAGENT or RESOURCE	SOURCE	IDENTIFIER
**Antibodies**		
Rabbit monoclonal anti-Galectin 7	Abcam	Cat#ab206435
Mouse monoclonal anti-COL1A1	Santa Cruz Biotechnology	Cat#sc-293182
Mouse monoclonal anti-ATP1B3	Santa Cruz Biotechnology	Cat#sc-135998
Rabbit monoclonal anti-CD44	Abcam	Cat#ab189524
Rabbit polyclonal anti-TP63	Abcam	Cat#ab97865
Fluorescein (FITC)-conjugated goat anti-rabbit IgG secondary antibody	Jackson Immunoresearch	Cat#111-095-003
Cy3-conjugated goat anti-mouse IgG secondary antibody	Jackson Immunoresearch	Cat#115-165-003
Mouse monoclonal anti-CD44	Cell Signaling Technologies	Cat#3570
Rabbit monoclonal anti-COL1A1	Cell Signaling Technologies	Cat#72026
Rabbit monoclonal anti-GAPDH	Cell Signaling Technologies	Cat#5174
Mouse monoclonal anti-a-tubulin	Merck Millipore	Cat#CP06
Anti-rabbit IgG, HRP-linked antibody	Cell Signaling Technologies	Cat#7074
Anti-mouse IgG, HRP-linked antibody	Cell Signaling Technologies	Cat#7076
Mouse monoclonal anti-CD3	Biogems	Cat#05112-20
Mouse monoclonal anti-CD28	Biogems	Cat#10312-20
Mouse monoclonal anti-CD4, PE-cy7	Biogems	Cat#06121-77
Rat monoclonal anti-FOXP3, APC	Invitrogen	Cat#17-4776-42
Mouse monoclonal anti-CD25, PE-cy7	Invitrogen	Cat#25-0259-42
Mouse monoclonal anti-CD3, PE	Invitrogen	Cat#12-0038-42
Mouse monoclonal anti-CD4, FITC	Invitrogen	Cat#11-0048-42
Mouse monoclonal anti-LAIR2 Alexa 488-conjugated antibody	R&D systems	Cat#IC2665G
**Chemicals, Peptides, and Recombinant Proteins**		
4’,6-diamidino-2-phenylindole (DAPI)	Sigma-Aldrich	Cat#D9542
Collagen, Type 1 solution from rat tail	Sigma-Aldrich	Cat#C3867
Recombinant Mouse CXCL2	PeproTech	212-12
Recombinant Human TGF-β	PeproTech	100-21
Recombinant Human Galectin 7	Abcam	Cat#ab62616
Recombinant Human CXCL8	PeproTech	Cat#P10145
Crystal violet	Sigma	Cat#C3886
**Critical Commercial Assays**		
Chromium Single Cell 3' Feature Barcode Library Kit v2	10X Genomics	Cat#PN-120234
Chromium Next GEM Single Cell 3' GEM, Library & Gel Bead Kit v2	10X Genomics	Cat#PN-120235
**Public Data**		
HNSCC scRNA-Seq data	Puram et al., 2017	GEO: GSE103322
HNSCC scRNA-Seq data	Kürten et al., 2021	GEO: GSE164690
TCGA-HNSCC	National Cancer Institute	https://portal.gdc.cancer.gov
Bulk RNA-seq data – HNSCC 97 cohort	Lohavanichbutr et al., 2013	GEO: GSE41613
Bulk RNA-seq data – HNSCC 74 cohort	Lohavanichbutr et al., 2013	GEO: GSE42743
Bulk RNA-seq data – HNSCC 270 cohort	Wichmann et al., 2015	GEO: GSE65858
**Software and Algorithms**		
R v4.1.0	R Core Team	https://www.r-project.org
10x Cell Ranger v2.1.1	10x Genomics	https://support.10xgenomics.com/ single-cell-gene-expression/software/pipelines/latest/ what-is-cell-ranger

Seurat v4.0.3	CRAN (R 4.1.0)	https://cran.r-project.org/
Harmony 1.0	Korsunsky et al., 2019	https://portals.broadinstitute.org/harmony/index.html
inferCNV 1.8.0	Bioconductor	bioconductor.org
Monocle2 2.20.0	Bioconductor	bioconductor.org
Monocle3 1.0.0	Bioconductor	bioconductor.org
MuSiC	Wang et al., 2019a	https://xuranw.github.io/MuSiC/articles/MuSiC.html
DNAcopy 1.66.0	Bioconductor	bioconductor.org
