# Supplementary material for: Single-cell transcriptome profiling of the stepwise progression of head and neck cancer
Source: Nat Commun. 2023 Feb 24;14:1055. doi: 10.1038/s41467-023-36691-x (PMC9958029; doi:10.1038/s41467-023-36691-x)
Supplement: Supplementary file 3 — Reporting Summary [file 41467_2023_36691_MOESM3_ESM.pdf]

nature portfolio

Corresponding author(s): Hyun Goo Woo

Last updated by author(s): Feb 8, 2023

Reporting Summary

Nature Portfolio wishes to improve the reproducibility of the work that we publish. This form provides structure for consistency and transparency in reporting. For further information on Nature Portfolio policies, see our Editorial Policies and the Editorial Policy Checklist.

Statistics

For all statistical analyses, confirm that the following items are present in the figure legend, table legend, main text, or Methods section.

n/a	Confirmed	
☐	☒	The exact sample size (*n*) for each experimental group/condition, given as a discrete number and unit of measurement
☐	☒	A statement on whether measurements were taken from distinct samples or whether the same sample was measured repeatedly
☐	☒	The statistical test(s) used AND whether they are one- or two-sided<br>*Only common tests should be described solely by name; describe more complex techniques in the Methods section.*
☐	☒	A description of all covariates tested
☐	☒	A description of any assumptions or corrections, such as tests of normality and adjustment for multiple comparisons
☐	☒	A full description of the statistical parameters including central tendency (e.g. means) or other basic estimates (e.g. regression coefficient) AND variation (e.g. standard deviation) or associated estimates of uncertainty (e.g. confidence intervals)
☐	☒	For null hypothesis testing, the test statistic (e.g. *F*, *t*, *r*) with confidence intervals, effect sizes, degrees of freedom and *P* value noted<br>*Give P values as exact values whenever suitable.*
☒	☐	For Bayesian analysis, information on the choice of priors and Markov chain Monte Carlo settings
☒	☐	For hierarchical and complex designs, identification of the appropriate level for tests and full reporting of outcomes
☐	☒	Estimates of effect sizes (e.g. Cohen's *d*, Pearson's *r*), indicating how they were calculated

Our web collection on statistics for biologists contains articles on many of the points above.

Software and code

Policy information about availability of computer code

Data collection	Single cell sequencing data were obtained using Chromium Single Cell 3' v2 Reagent Kits and the HiSeq 4000 sequencer. Sequencing data were aligned to the human reference genome (GRCh38) and processed using CellRanger 2.1.1 (10x Genomics).
Data analysis	Statistical analysis was performed using the R software (version 3.4.0; Vienna, Austria).

For manuscripts utilizing custom algorithms or software that are central to the research but not yet described in published literature, software must be made available to editors and reviewers. We strongly encourage code deposition in a community repository (e.g. GitHub). See the Nature Portfolio guidelines for submitting code & software for further information.

Data

Policy information about availability of data

All manuscripts must include a data availability statement. This statement should provide the following information, where applicable:

- Accession codes, unique identifiers, or web links for publicly available datasets
- A description of any restrictions on data availability
- For clinical datasets or third party data, please ensure that the statement adheres to our policy

The raw and processed data generated in this study have been deposited in the database under accession code GSE181919 [https://www.ncbi.nlm.nih.gov/geo/query/acc.cgi?acc=GSE181919]. The following publicly available datasets were used in the study: GSE103322 [https://www.ncbi.nlm.nih.gov/geo/query/acc.cgi?acc=GSE103322] and GSE164690 [https://www.ncbi.nlm.nih.gov/geo/query/acc.cgi?acc=GSE164690], scRNA-seq of HNSCC; GSE41613 [https://

www.ncbi.nlm.nih.gov/geo/query/acc.cgi?acc=GSE41613], GSE42743 [https://www.ncbi.nlm.nih.gov/geo/query/acc.cgi?acc=GSE42743], GSE65858 [https://www.ncbi.nlm.nih.gov/geo/query/acc.cgi?acc=GSE65858], and TCGA-HNSCC (https://portal.gdc.cancer.gov/), bulk transcriptome profiles of HNSCC. Source data are provided with this paper.

Human research participants

Policy information about studies involving human research participants and Sex and Gender in Research.

Reporting on sex and gender	Patient sex information was not considered in study design.
Population characteristics	See above.
Recruitment	A total of 37 tissue specimens were obtained from 23 patients (HNSCC cohorts from Ajou University Hospital).
Ethics oversight	All the experiments with patient samples were performed under the approval of the Ajou University Institutional Review Board, using the approved protocol AJIRB-BMR-SMP-18-150 with the written informed consent of all patients. No compensation was provided for patient participant.

Note that full information on the approval of the study protocol must also be provided in the manuscript.

Field-specific reporting

Please select the one below that is the best fit for your research. If you are not sure, read the appropriate sections before making your selection.

☒ Life sciences ☐ Behavioural & social sciences ☐ Ecological, evolutionary & environmental sciences

For a reference copy of the document with all sections, see nature.com/documents/nr-reporting-summary-flat.pdf

Life sciences study design

All studies must disclose on these points even when the disclosure is negative.

Sample size	No sample size calculation was performed to pre-determine sample sizes. A total of 37 tissue specimens were obtained from 23 patients (HNC cohorts from Ajou University Hospital), including tissues from non-tumoral surrounding normal tissue (NL, n = 9), leukoplakia (LP, n = 4), primary cancer (CA, n = 20), and metastatic tumors in lymph nodes (LN, n = 4). The number of samples in this study is comparable to the other studies.
Data exclusions	Data were not excluded from analysis
Replication	All experiments replicated at least 3 times, and details are provided in corresponding figure legend.
Randomization	No randomization of the samples were required, as all samples were analyzed immediately after surgical resection.
Blinding	Blinding is not possible, because the groups were designated based on the pathological findings. Computational analyses were applied equally to all conditions and replicates

Reporting for specific materials, systems and methods

We require information from authors about some types of materials, experimental systems and methods used in many studies. Here, indicate whether each material, system or method listed is relevant to your study. If you are not sure if a list item applies to your research, read the appropriate section before selecting a response.

Materials & experimental systems

n/a	Involved in the study	
☐	☒	Antibodies
☐	☒	Eukaryotic cell lines
☒	☐	Palaeontology and archaeology
☐	☒	Animals and other organisms
☒	☐	Clinical data
☒	☐	Dual use research of concern

Methods

n/a	Involved in the study	
☒	☐	ChIP-seq
☐	☒	Flow cytometry
☒	☐	MRI-based neuroimaging

Antibodies

Antibodies used	Rabbit monoclonal anti-Galectin 7 Abcam Cat#ab206435<br>Mouse monoclonal anti-COL1A1 (1:40) Santa Cruz Biotechnology Cat#sc-293182<br>Mouse monoclonal anti-ATP1B3 Santa Cruz Biotechnology Cat#sc-135998<br>Rabbit monoclonal anti-CD44 (1:1000) Abcam Cat#ab189524<br>Rabbit polyclonal anti-TP63 (1:200) Abcam Cat#ab97865<br>Fluorescein (FITC)-conjugated goat anti-rabbit IgG secondary antibody Jackson Immunoresearch Cat#111-095-003<br>Cy3-conjugated goat anti-mouse IgG secondary antibody Jackson Immunoresearch Cat#115-165-003<br>Mouse monoclonal anti-CD44 Cell Signaling Technologies Cat#3570<br>Rabbit monoclonal anti-COL1A1 Cell Signaling Technologies Cat#72026<br>Rabbit monoclonal anti-GAPDH Cell Signaling Technologies Cat#5174<br>Mouse monoclonal anti-a-tubulin Merck Millipore Cat#CP06<br>Anti-rabbit IgG, HRP-linked antibody Cell Signaling Technologies Cat#7074<br>Anti-mouse IgG, HRP-linked antibody Cell Signaling Technologies Cat#7076<br>Mouse monoclonal anti-CD3 Biogems Cat#05112-20<br>Mouse monoclonal anti-CD28 Biogems Cat#10312-20<br>Mouse monoclonal anti-CD4, PE-cy7 Biogems Cat#06121-77<br>Rat monoclonal anti-FOXP3, APC Invitrogen Cat#17-4776-42<br>Mouse monoclonal anti-CD25, PE-cy7 Invitrogen Cat#25-0259-42<br>Mouse monoclonal anti-CD3, PE Invitrogen Cat#12-0038-42<br>Mouse monoclonal anti-CD4, FITC Invitrogen Cat#11-0048-42<br>Mouse monoclonal anti-LAIR2 Alexa 488-conjugated antibody R&D systems Cat#IC2665G
Validation	All antibodies used are commercially available and were validated by manufacturer for that application. All antibodies were tested in the laboratory and titrated prior to all experiments.<br><br>https://www.abcam.com/galectin-7-antibody-epr19903-ab206435.html<br>https://www.scbt.com/ko/p/col1a1-antibody-3g3<br>https://www.scbt.com/ko/p/na-k-atpase-beta3-antibody-46<br>https://www.abcam.com/cd44-antibody-epr18668-ab189524.html<br>https://www.abcam.com/p63-antibody-ab97865.html<br>https://www.jacksonimmuno.com/catalog/products/111-095-003<br>https://www.jacksonimmuno.com/catalog/products/115-165-003<br>https://www.cellsignal.com/products/primary-antibodies/cd44-156-3c11-mouse-mab/3570<br>https://www.cellsignal.com/products/primary-antibodies/col1a1-e8f4l-xp-rabbit-mab/72026<br>https://www.cellsignal.com/products/primary-antibodies/gapdh-d16h11-xp-rabbit-mab/5174<br>https://www.sigmaaldrich.com/KR/ko/product/mm/cp06<br>https://www.cellsignal.com/products/secondary-antibodies/anti-rabbit-igg-hrp-linked-antibody/7074<br>https://www.cellsignal.com/products/secondary-antibodies/anti-mouse-igg-hrp-linked-antibody/7076<br>https://www.bio-gems.com/anti-mouse-cd3-apc-cy7.html<br>https://www.bio-gems.com/anti-mouse-cd28-purified.html<br>https://www.bio-gems.com/flow-cytometry/conjugated-antibodies/anti-human-cd4-pe-cy7.html<br>https://www.thermofisher.com/antibody/product/FOXP3-Antibody-clone-PCH101-Monoclonal/17-4776-42<br>https://www.thermofisher.com/antibody/product/CD25-Antibody-clone-BC96-Monoclonal/25-0259-42<br>https://www.thermofisher.com/antibody/product/CD3-Antibody-clone-UCHT1-Monoclonal/12-0038-42<br>https://www.thermofisher.com/antibody/product/CD4-Antibody-clone-OKT4-OKT-4-Monoclonal/11-0048-42<br>https://www.rndsystems.com/products/human-lair2-alexa-fluor-488-conjugated-antibody-319701_ic2665g

Eukaryotic cell lines

Policy information about cell lines and Sex and Gender in Research

Cell line source(s)	FaDu cells were obtained from American Type Culture Collection (Manassas, VA, USA) and cultured in Minimum Essential Medium (MEM; Welgene, Gyeongsangbuk-do, South Korea). MSKQLL1, SCCQLL1, SCC15, and SCC25 were kindly provided by Prof. Se-Heon Kim (Yonsei University, Korea). MSKQLL1, SCC15, and SCC25 cells were maintained in Dulbecco's modified Eagle's Medium/Nutrient Mixture F-12 (DMEM/F12; Welgene). SCCQLL1 were cultured in Minimum Essential Medium (MEM; Welgene). All growth media were supplemented with 10 % fetal bovine serum, and cells were cultured at 37°C in a humidified atmosphere containing 5 % CO2.
Authentication	None of the cell lines have been authenticated.
Mycoplasma contamination	Cell lines were not tested for mycoplasma contamination but no indication of contamination was observed.
Commonly misidentified lines<br>(See ICLAC register)	None of the commonly misidentified lines were used.

Animals and other research organisms

Policy information about studies involving animals; ARRIVE guidelines recommended for reporting animal research, and Sex and Gender in Research

Laboratory animals	CD4+ T cells were isolated from C57/BL6 mouse lymph nodes and spleen using mouse CD3+ T cell enrichment columns (R&D Systems, MN, USA) and CD4+ MicroBeads (Miltenyi Biotec) according to the manufacturer's instructions.
Wild animals	No wild animals were used in this study.
Reporting on sex	See above
Field-collected samples	No field-collected samples were used in this study
Ethics oversight	The study was performed under the approval of the Institutional Animal Care and Use Committee.

Note that full information on the approval of the study protocol must also be provided in the manuscript.

Flow Cytometry

Plots

Confirm that:

☒ The axis labels state the marker and fluorochrome used (e.g. CD4-FITC).

☒ The axis scales are clearly visible. Include numbers along axes only for bottom left plot of group (a 'group' is an analysis of identical markers).

☒ All plots are contour plots with outliers or pseudocolor plots.

☒ A numerical value for number of cells or percentage (with statistics) is provided.

Methodology

Sample preparation	Human peripheral blood mononuclear cells (PBMCs, n = 1x10^7) were isolated from five HNSCC patients using a SepMate PBMC isolation tube containing Ficoll (STEMCELL Technologies, Canada). The PMBCs were cultured on Collagen type 1 (10 μg/cm2) coated or non-coated plate for 36 hr. After cell collection, the cells were stained with monoclonal antibodies for CD3, CD4, CD25, FOXP3 (Invitrogen, Carlsbad, CA), and LAIR2 (R&D system, Minneapolis, MN). Then, the intracellular staining was performed using the Foxp3/Transcription Factor Staining Buffer set (Invitrogen Carlsbad, CA). Flow cytometry was performed using a Becton Dickinson FACSAria III (BD Biosciences, Franklin Lakes, NJ) and analyzed using FlowJo software (Ashland, OR).
Instrument	Flow cytometry was performed using a Becton Dickinson FACSAria III (BD Biosciences, Franklin Lakes, NJ)
Software	FACS Data were analyzed using FlowJo software (Ashland, OR).
Cell population abundance	Human peripheral blood mononuclear cells (PBMCs, n = 1x10^7) were isolated from five HNSCC patients using a SepMate PBMC isolation tube containing Ficoll (STEMCELL Technologies, Canada).
Gating strategy	Cells were gated according to their forward and side scatter.

☒ Tick this box to confirm that a figure exemplifying the gating strategy is provided in the Supplementary Information.
